# Cholesterol esterification inhibition and imatinib treatment synergistically inhibit growth of BCR-ABL mutation-independent resistant chronic myelogenous leukemia

**DOI:** 10.1371/journal.pone.0179558

**Published:** 2017-07-18

**Authors:** Shovik Bandyopadhyay, Junjie Li, Elie Traer, Jeffrey W. Tyner, Amy Zhou, Stephen T. Oh, Ji-Xin Cheng

**Affiliations:** 1 Department of Biological Sciences, Purdue University, West Lafayette, Indiana, United States of America; 2 Weldon School of Biomedical Engineering, Purdue University, West Lafayette, Indiana, United States of America; 3 Knight Cancer Institute, Oregon Health & Science University, Portland, Oregon, United States of America; 4 Department of Cell, Developmental & Cancer Biology, Oregon Health & Science University, Portland, Oregon, United States of America; 5 Division of Hematology, Washington University School of Medicine, St. Louis, Missouri, United States of America; 6 Center for Cancer Research, Purdue University, West Lafayette, Indiana, United States of America; Florida International University, UNITED STATES

## Abstract

Since the advent of tyrosine kinase inhibitors (TKIs) such as imatinib, nilotinib, and dasatinib, chronic myelogenous leukemia (CML) prognosis has improved greatly. However, ~30–40% of patients develop resistance to imatinib therapy. Although most resistance is caused by mutations in the BCR-ABL kinase domain, 50–85% of these patients develop resistance in the absence of new mutations. In these cases, targeting other pathways may be needed to regain clinical response. Using label-free Raman spectromicroscopy, we evaluated a number of leukemia cell lines and discovered an aberrant accumulation of cholesteryl ester (CE) in CML, which was found to be a result of BCR-ABL kinase activity. CE accumulation in CML was found to be a cancer-specific phenomenon as untransformed cells did not accumulate CE. Blocking cholesterol esterification with avasimibe, a potent inhibitor of acyl-CoA cholesterol acyltransferase 1 (ACAT-1), significantly suppressed CML cell proliferation in Ba/F3 cells with the BCR-ABL^T315I^ mutation and in K562 cells rendered imatinib resistant without mutations in the BCR-ABL kinase domain (K562R cells). Furthermore, the combination of avasimibe and imatinib caused a profound synergistic inhibition of cell proliferation in K562R cells, but not in Ba/F3^T315I^. This synergistic effect was confirmed in a K562R xenograft mouse model. Analysis of primary cells from a BCR-ABL mutation-independent imatinib resistant patient by mass cytometry suggested that the synergy may be due to downregulation of the MAPK pathway by avasimibe, which sensitized the CML cells to imatinib treatment. Collectively, these data demonstrate a novel strategy for overcoming BCR-ABL mutation-independent TKI resistance in CML.

## Introduction

Development of imatinib (IM) therapy has improved the prognosis of chronic myelogenous leukemia (CML) considerably. However, ~30–40% of patients fail to respond optimally to IM treatment.[1] The majority of research on imatinib resistance in CML has been focused on identifying methods to overcome resistance driven by BCR-ABL kinase domain mutations through the use of second and third generation tyrosine kinase inhibitors (TKIs), including dasatinib, nilotinib, ponatinib, and others. Much less attention has been given to BCR-ABL resistance in the absence of mutations, which accounts for as many as 50–85% of clinically resistant patients treated with imatinib.[2] Additionally, treatment with TKIs has been documented to have significant safety issues. As many as 31% of patients have to discontinue imatinib treatment before a complete remission is achieved due to imatinib-intolerance.[3] Furthermore, almost 60% of patients relapse within 1–2 years of imatinib discontinuation.[4] Thus, there is a need for a safer, targeted approach to treat IM-resistant CML independent of BCR-ABL point mutations that achieves a deep, sustainable cytogenetic response.

One major mechanism of resistance in CML independent of BCR-ABL kinase domain mutations is the activation of alternate signaling pathways.[5,6] For example, mitogen-activated protein kinase (MAPK)/Protein Kinase C (PKC) pathway activation has been identified as a major driver of BCR-ABL mutation-independent imatinib resistance.[7] Imatinib alone is inherently incapable of rendering deep molecular responses in these cases. It also makes the rationale for imatinib discontinuation less clear if patients are unable to achieve complete cytogenetic remission.

Alongside the aberrant signaling characteristics of cancerous growth, many cancer cells display altered lipid metabolism.[8,9] For example, elevated de novo lipogenesis has been well characterized in many cancers.[10,11] Aberrant cholesterol metabolism, such as accumulation of cholesteryl ester (CE) has been found in breast cancer,[12] leukemia,[13] glioma,[14] pancreatic cancer,[15] and prostate cancer.[16] Targeting cholesterol esterification by inhibition of the enzyme acetyl-CoA cholesterol acyltransferase 1 (ACAT-1) has been shown to reduce proliferation in solid tumors [16–18] as well as lymphocytic leukemia.[13] Despite these advances, lipid metabolism in IM-resistant CML has never been studied.

In this report, we show that CML cells accumulate high levels of CE, and that this phenomenon is related to BCR-ABL kinase activity, as non-malignant hematopoietic cells as well as AML cells do not exhibit high levels of CE. Importantly, CML cells rendered IM resistant by BCR-ABL independent mechanisms retain this phenotype of high CE levels. By using a combination of imatinib and avasimibe, an inhibitor of ACAT-1, we demonstrate a synergistic effect in suppressing cell proliferation in imatinib resistant CML cells, but not in normal cells or imatinib sensitive CML cells. Mechanistically, we show the synergy is in part due to downregulation of the MAPK pathway by avasimibe, which is activated in IM resistant CML. Collectively, this study presents a novel strategy for overcoming TKI resistance through targeting altered cholesterol metabolism.

## Materials and methods

### Cell lines

MOLM14, RCH-ACV, K562, and Kasumi-2 cell lines were obtained from DSMZ and maintained in RPMI medium supplemented with 10% fetal bovine serum, 2 mM L-glutamine, and 0.5% penicillin/streptomycin. Ba/F3 and 32D cells were originally purchased from American Type Culture Collection (ATCC). Ba/F3 and 32D cells overexpressing empty vector, BCR-ABL, BCR-ABL^T315I^ or BCR-ABL^kinase dead^ were generated as previously described and maintained in the same medium as mentioned above. [19,20] K562R cell lines, which display IM resistance in the absence of BCR-ABL mutations, were initially generated by culturing naïve K562 cells with FGF2 and imatinib, as described previously.[21] Resistant K562R cells were maintained in 0.5–1 μM imatinib. Multiple K562R cell lines were generated and tested for similar behavior. Sequencing of the BCR-ABL and FGFR3 genes in K562R revealed no mutations.

### Inhibitors and reagents

Imatinib (free base) for use in the in-vitro assays was purchased from ChemieTek and dissolved in DMSO. Avasimibe and imatinib mesylate (for in vivo experiments) were purchased from SelleckChem. Imatinib mesylate was dissolved in water, while Avasimibe was always dissolved in DMSO.

### Cell viability assays

Cells were plated at 4000 cells per well on Day 0. Cell viability after treatment for 72 hours was measured by intensity of luminescent signal as read by a SpectraMax M5 Plate Reader using the ATP assay Cell Titer Glo reagent from Promega. Luminescent signal for each condition was then normalized to the wells with no inhibitor. Control and treatment wells were always treated with DMSO to equalize total volume across all wells. Combination index was analyzed by the Chou-Talalay method using CompuSyn software.[22]

### Mass cytometry

Single-cell protein analysis was performed using a CyTOF2 instrument at the Washington University School of Medicine Immunomonitoring Laboratory according to previously published procedures.[23] All metal-conjugated antibodies were purchased from Fluidigm. Cells were treated with 1μM imatinib for 30 minutes or 10μM avasimibe for 4 hours. The full antibody panel used for analysis of patient samples is detailed in S1 Table. Data analysis was performed using Cytobank as described previously,[23] with specific gating strategies detailed in S4 Fig. Further analysis was performed using viSNE.[24] Details on gating of viSNE figures can be found in S6 Fig.

### Patient samples

All patient samples were obtained with written consent according to a protocol approved by the Washington University Human Studies Committee (WU no. 01–1014). All CML patient samples had wild-type BCR-ABL (data not shown).

### Mouse models

All animal experiments were conducted following a protocol approved by the Purdue Animal Care and Use Committee (PACUC). 4–6 week old athymic nude mice from Harlan Laboratories were subcutaneously inoculated with 5x10^6^ K562R cells per mouse. Mice were anesthetized using isoflurane inhalation when injection was performed. Every effort was made to minimize suffering. Tumor volumes were measured using a caliper and calculated as 1/2 × L × W^2^, where L stands for the length, and W for the width in mm. Mice were divided into four groups (n = 8 each group) once average tumor volume reached approximately 100 mm^3^. One group received only DMSO vehicle, one group received IM+ DMSO, one group received avasimibe alone, and the fourth group received a combination of avasimibe and IM. Avasimibe was administered daily by intraperitoneal injection at a dose of 7.5 mg/kg, and IM was administered daily by oral gavage at a dose of 70 mg/kg. Treatment was discontinued when one xenograft reached a volume of 2000 mm^3^ or when the tumor is interfering with movement, whichever occurs first. Mice were euthanized by cervical dislocation following deep anesthesia induced by isoflurane, as approved by PACUC protocol, and the xenografts were harvested. Data was analyzed using the Student’s T-Test.

### Raman spectromicroscopy

Confocal Raman spectral analysis from individual lipid droplets (LDs) were performed as described previously [25]. A 5-picosecond laser at 707 nm was used as excitation beam for Raman spectral acquisition. Acquisition time for a typical spectrum from individual LDs was 20 s, with the beam power maintained around 15 mW at sample. For each specimen, at least 10 spectra from individual LDs in different locations or cells were obtained. The spectra were analyzed using software Origin 8.5. The background was removed manually, and peak height was measured.

### Image acquisition and processing

Stimulated Raman scattering (SRS) microscopy was performed with two femto-second laser system. Specifically, a Ti:Sapphire laser (Chameleon Vision, Coherent) with up to 4W (80 MHz, ~140 fs pulse width) pumps an optical parametric oscillator (OPO, Chameleon Compact, Angewandte Physik & Elektronik GmbH). The pump and Stokes beams were tuned to 830 nm and 1090 nm, respectively. The pump and Stokes pulse trains were collinearly overlapped and directed into a laser-scanning microscope (FV300, Olympus). A 60X water-immersion objective lens (UPlanSApo, Olympus) was used to focus the laser into a sample. An oil condenser of 1.4 numerical aperture (NA) was used to collect the signal in a forward direction. The typical acquisition time for a 512 x 512 pixels SRL image was 1.12 second. Images were processed using ImageJ. To quantify the LD area fraction, the LDs were picked up by applying an intensity threshold. This same threshold was applied for each sample for one experiment. The percentage of LDs area out of the total cellular area was measured.

## Results

### Abnormal CE accumulation in chronic myeloid leukemia (CML) is driven by BCR-ABL

**Fig 1:**
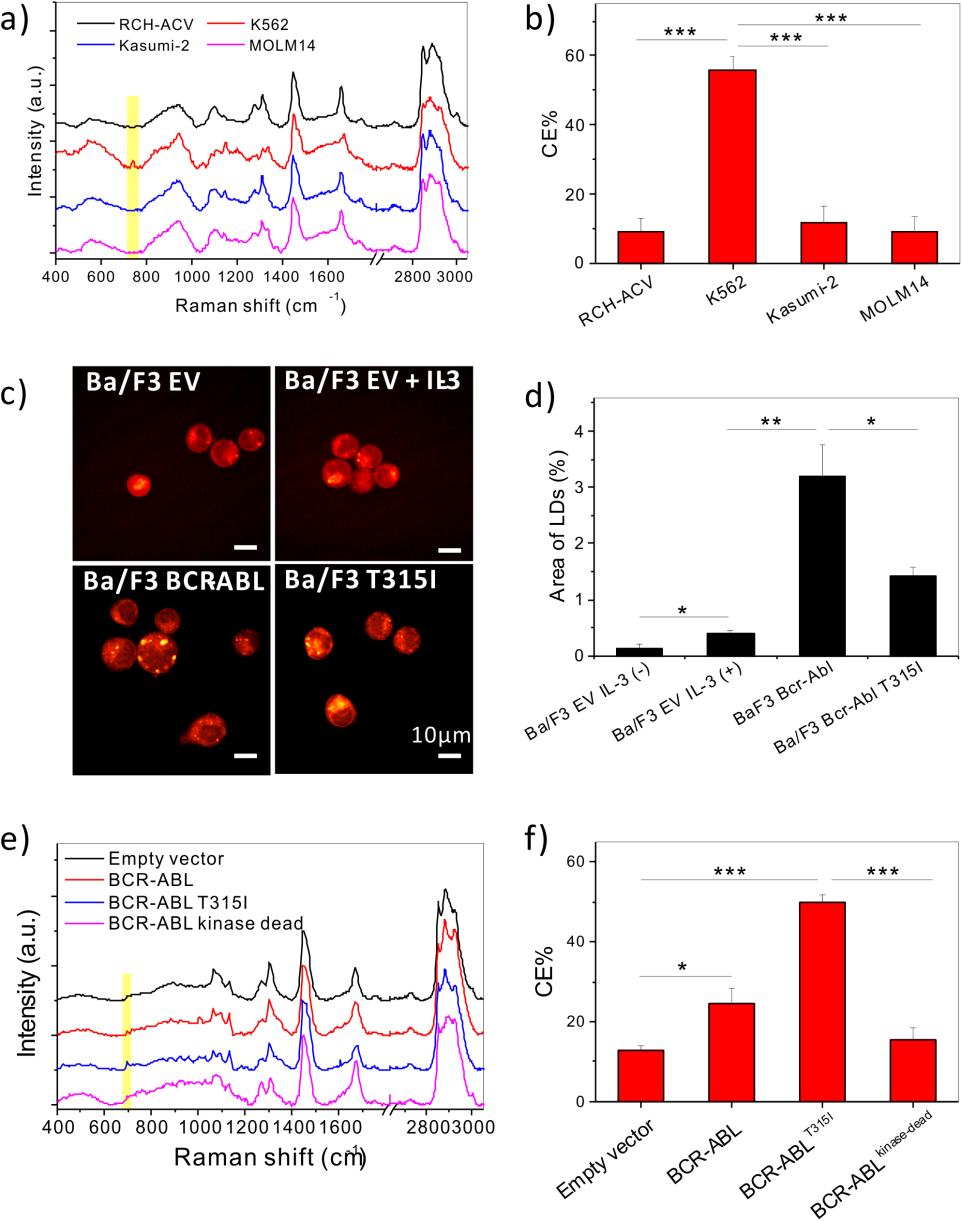
CE accumulation in CML. (a) Raman Spectra acquired from LDs of four leukemia cell lines, including RCH-ACV (ALL), K562 (CML), Kasumi-2 (ALL), and MOLM14 (AML).(b) Quantification of CE% out of total lipids in LDs in four leukemia cell lines. (c) Representative SRS images of Ba/F3 Cells overexpressing empty vector treated with or without IL-3, BCR-ABL^WT^, or BCR-ABL^T315I^ cells. (d) Quantification of LD amount by area fraction analysis from SRS images. (e) Raman spectra of LDs in 32D cells overexpressing empty vector, BCR-ABL, BCR-ABL^T315I^, or BCR-ABL^kinase-dead^. (f) Quantification of CE% in LDs from 32D cells. For quantitative analysis, all the results are shown as means + SEM, n = 4~6. Two-way student t test was used for statistical analysis, * *p* < 0.05, ** *p* < 0.01, *** *p* < 0.001.

To characterize the lipid metabolism in leukemia cells, Raman spectral analysis was performed on a variety of well-characterized leukemia cell lines, including MOLM14 (AML), RCH-ACV (ALL), Kasumi-2 (ALL), and K562 (CML) cells. An abnormal accumulation of CE was identified in K562 cells, as evidenced by the peak at Raman shift of 702cm^-1^ from cholesterol ring vibration [16] (Fig 1A). Quantitative analysis revealed a 50% level of CE in the lipid droplets of K562 cells, but only around 10% in the other leukemia cell lines examined (Fig 1B).

Considering the correlation between BCR-ABL activation and CE accumulation in CML, we hypothesized that BCR-ABL drives CE accumulation. To assess whether BCR-ABL was necessary and sufficient to cause CE accumulation, a murine interleukin-3 dependent pro-B cell line Ba/F3 was used. Ba/F3 cells overexpressing BCR-ABL^WT^, BCR-ABL^T315I^, or empty vector (control) were subjected to SRS imaging to visualize LD accumulation in the three cell lines (Fig 1C). Ba/F3 cells transduced with empty vector showed no accumulation of LDs, regardless of whether they were stimulated with IL-3 for 48 hours. On the other hand, Ba/F3 BCR-ABL^WT^ and Ba/F3 BCR-ABL^T315I^ cells had LD accumulation even without IL-3 stimulation (Fig 1C and 1D). Through Raman spectral analysis, these LDs were found to be mainly composed of CE (65–75%) (S1A and S1B Fig). The Ba/F3 control cells could not be spectrally analyzed because there were no detectable LDs. Consistently, in another mouse bone marrow derived cell line, 32D cells overexpressing BCR-ABL or BCR-ABL^T315I^ accumulated significantly more CE than empty vector controls. In contrast, 32D cells overexpressing BCR-ABL^kinase-dead^ did not induce accumulation of CE compared to empty vector control (Fig 1E and 1F), indicating BCR-ABL kinase activity is necessary for CE accumulation. SRS Imaging of 32D cells revealed that BCR-ABL kinase activity was required for LD accumulation in these cells as well (S1C Fig). Treatment with avasimibe was sufficient to remove CE in Ba/F3 BCR-ABL^WT^ cells (S1D and S1E Fig), suggesting the potential of targeting cholesterol metabolism in BCR-ABL driven CML.

### Avasimibe resensitizes BCR-ABL mutation-independent imatinib-resistant CML in vitro

**Fig 2:**
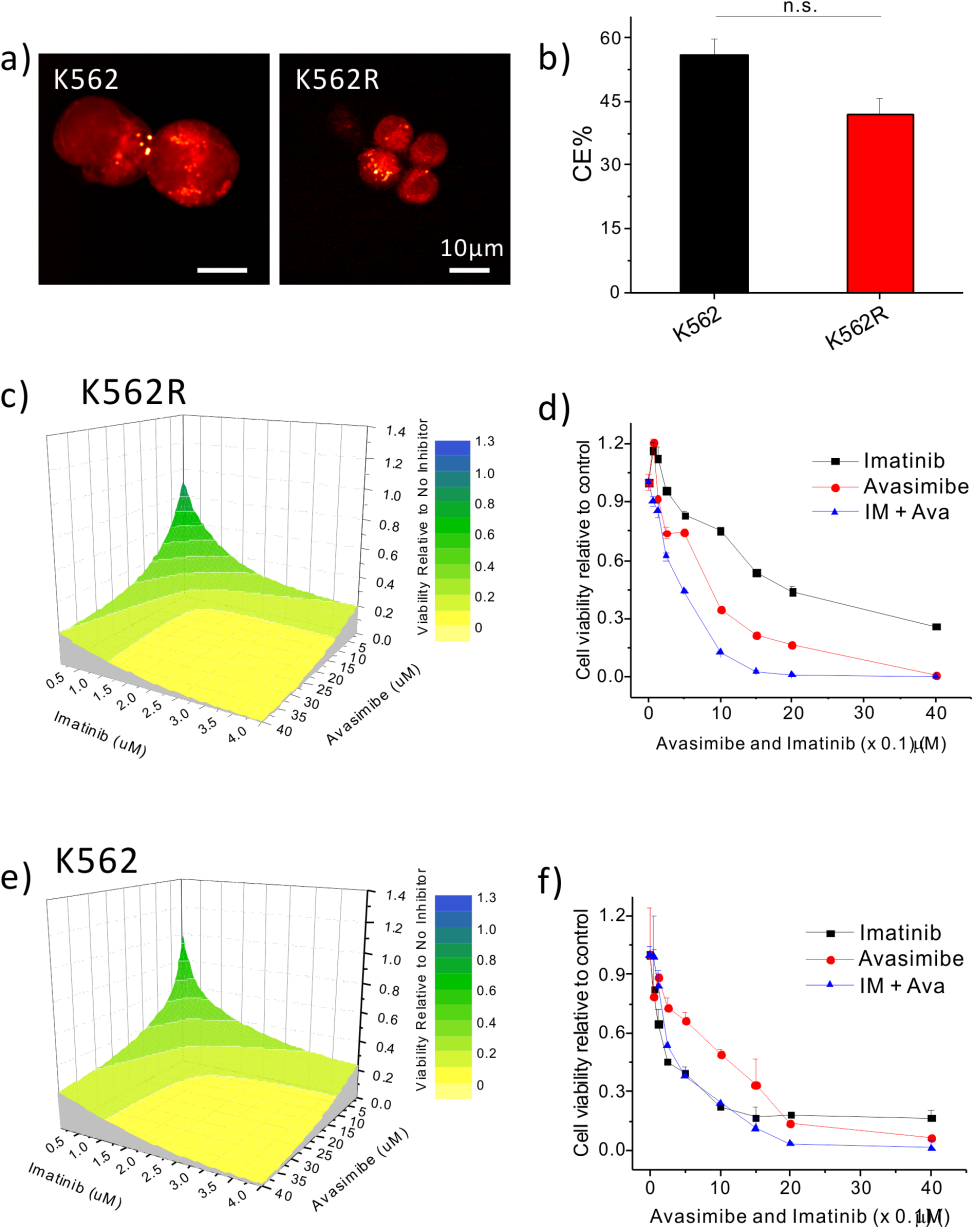
Imatinib and avasimibe show a significant synergy in inhibiting viability of K562R cells. (a) Representative SRS images of K562 and K562R cells. (b) Quantification of CE% in LDs of K562 and K562R cells. The results are shown as means + SEM, n = 6. Two-way student t test was used for statistical analysis; n.s. indicates no significance. (c) 3D contour plot with colormap (d) linear plot of K562R cells treated with imatinib, avasimibe, or combination of imatinib and avasimibe at a molar concentration ratio of 1: 10 (IM: Ava) for 72 hours. (e) 3D contour plot with colormap (f) linear plot of K562 cells treated with imatinib, avasimibe, or combination of imatinib and avasimibe at a molar concentration ratio of 1: 10 (IM: Ava) for 72 hours.Viability was measured using the Cell Titer Glo assay, with all viabilities normalized to no inhibitor treatment group. The results are shown as means + SEM, n = 3.

To test whether CE accumulation occurs in BCR-ABL mutation-independent IM resistant CML, the K562R cell line was established.[21] This cell line was rendered imatinib-resistant by BCR-ABL independent mechanisms, and is grown without loss of viability in 1μM imatinib. SRS imaging was used to visualize the LDs in individual K562R cells, as compared to K562. SRS imaging showed noticeable LD accumulation in both cell lines (Fig 2A). Raman spectral analysis on individual lipid droplets confirmed a high percentage of CE in their LDs (Fig 2B).

To test whether avasimibe could overcome imatinib resistance in CML, K562R cells displaying BCR-ABL mutation-independent resistance were treated with avasimibe and imatinib. The combination of avasimibe and imatinib at a 10:1 fixed concentration ratio in K562R cells yielded a significant reduction in cell viability at all concentrations tested (Fig 2C and 2D). The combination index (CI) as defined by the Chou-Talalay method[22] indicated a strong synergistic effect between avasimibe and imatinib (S2 Fig). This synergy was unique to BCR-ABL mutation-independent imatinib resistant K562R cells, as the combination of avasimibe and imatinib did not show a synergistic effect in naïve K562 cells (Fig 2E and 2F) or BCR-ABL dependent imatinib resistant Ba/F3 BCR-ABL^T315I^ cells (S2 and S3 Figs).

### Avasimibe and imatinib synergistically reduce tumor growth in a xenograft mouse model

**Fig 3:**
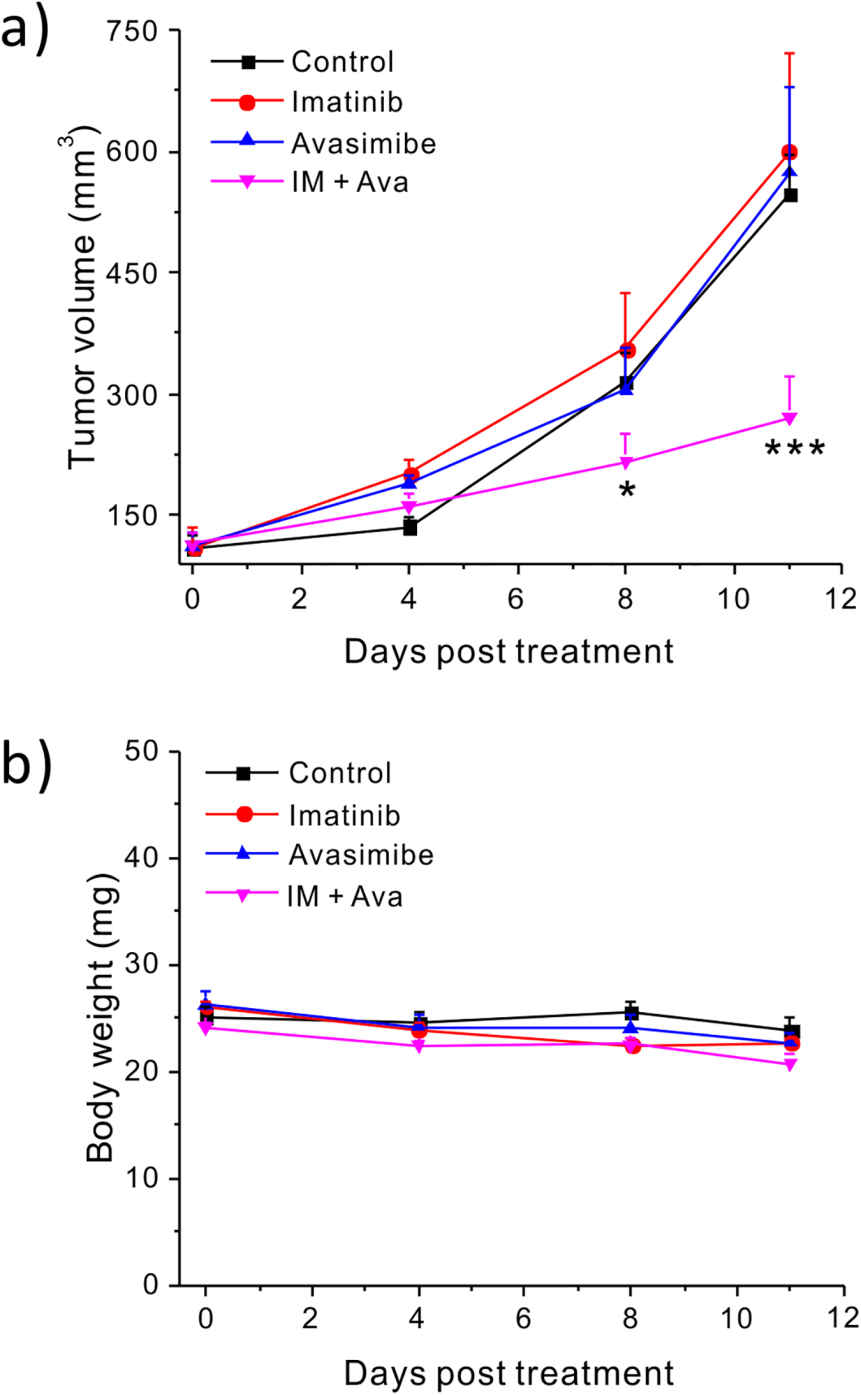
Imatinib and avasimibe synergistically suppress K562R xenograft tumor growth in athymic nude mice. (a) Tumor volume (mm^3^) measured by a caliper over the course of treatment for the four treatment groups. (b) Body weight (g) of the mice throughout the course of treatment. The results are shown as means + SEM, n = 8. One-way student t test was used for statistical analysis, * *p* < 0.05, *** *p* < 0.001

To confirm the synergy between avasimibe and imatinib *in vivo*, we used a xenograft mouse model. The combination treatment significantly (p<0.001) reduced tumor growth as compared to the control (DMSO), imatinib, or avasimibe alone treated groups (Fig 3A). Moreover, no significant treatment related body weight loss was observed (Fig 3B). These data suggest that a combination of avasimibe and imatinib could be a promising therapeutic strategy to treat imatinib-resistant CML without BCR-ABL kinase domain mutations.

### Avasimibe induces downregulation of the MAPK pathway

**Fig 4:**
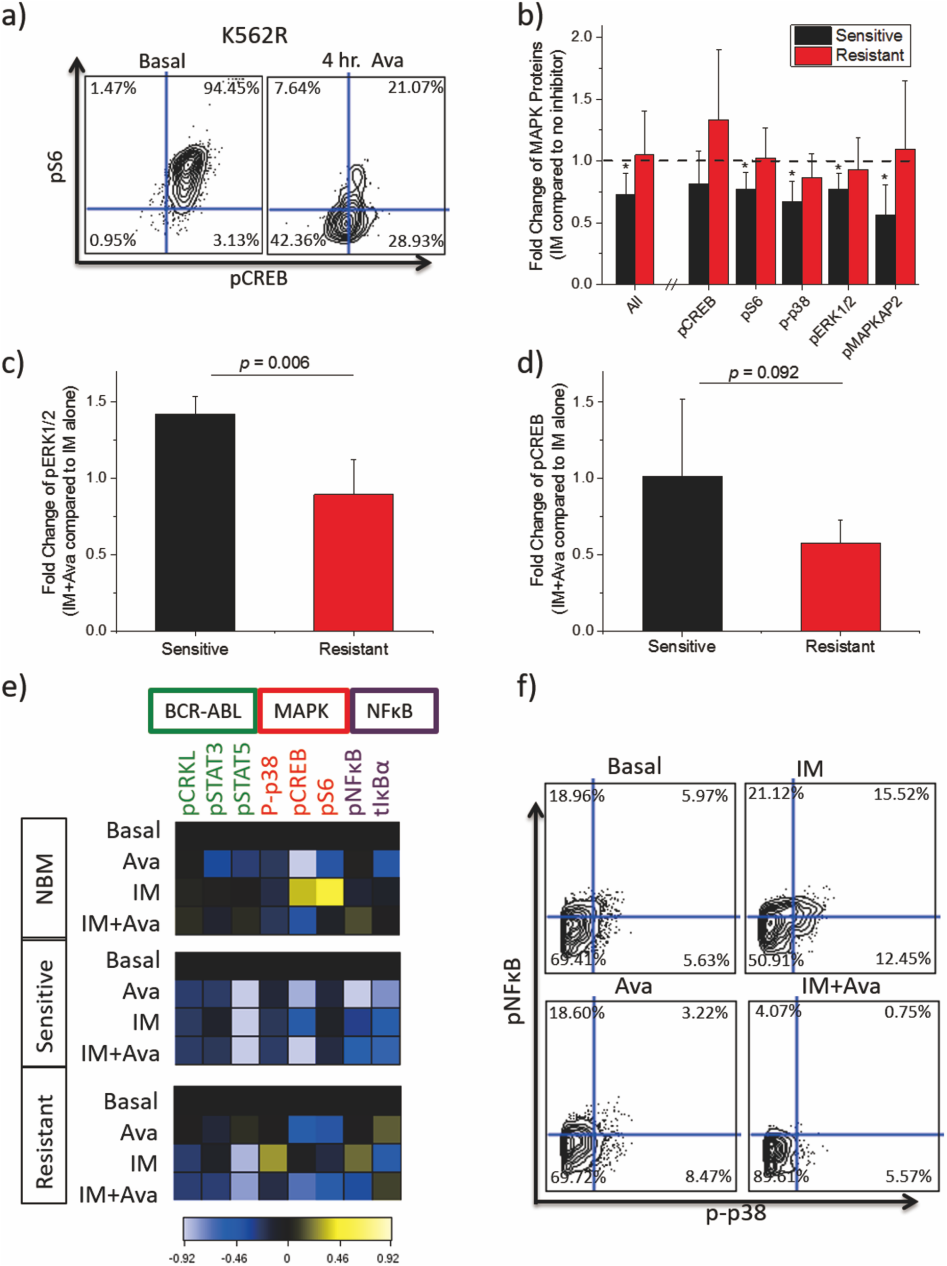
Avasimibe downregulates the MAPK pathway. (a) Contour biaxials of pS6 (y-axis) and pCREB (x-axis) gated on pCRKL+ cells collected by CyTOF in K562R cells. Cells were treated for 0 or 4 hours with10μM avasimibe. (b) Effect of 30 minute 5μM imatinib treatment on sensitive and resistant patients normalized to the basal condition on the pooled MAPK pathway proteins together (All) and individually. Error bars represent standard deviation of fold change in each group of patients. T-tests were conducted comparing fold change in resistant patients to sensitive patients (p-values shown) and for general reduction in phosphorylation (*- p<0.05) (c-d) Bar graphs showing fold change of median protein expression after 10μM avasimibe and combination therapy normalized to 5μM imatinib in resistant and sensitive CML patients (n = 4 for all groups except SCML3 was omitted in the pERK group because zero pERK signal was observed). Imatinib treatment was for thirty minutes while avasimibe treatment was for four hours. (e) Heatmaps of CyTOF screens of non-lymphoid CD34^+^ CD38^−^ cells from cryopreserved bone marrow from a normal patient (top), cryopreserved bulk PBMCs from an imatinib-sensitive patient (middle), and cryopreserved bulk PBMCs from an imatinib-resistant patient without a BCR-ABL kinase domain mutation (bottom). Cells were treated with no inhibitor, 1μM imatinib, 10μM avasimibe, or imatinib plus avasimibe at the same concentrations. Imatinib stimulation was done for 30 minutes, while avasimibe stimulation was done for four hours. Heatmap tile color represents arcsinh ratio of medians normalized to the basal condition for each patient, see Bendall et al. 2011[23] for details. (f) Biaxials of p-p65/NFκB on the x-axis versus p-p38/MAPK on the y-axis in Lin^-^ CD34^+^ CD38^−^ collected by CyTOF from the resistant patient. Each plot represents one of the four stimulation conditions: basal (top left), imatinib (top right), avasimibe (bottom left), and imatinib + avasimibe (bottom right). The contour represents cell density.

To understand the mechanism of drug synergy, signaling responses to avasimibe in K562R cells were examined via mass cytometry (CyTOF). Our results demonstrated sensitivity of K562R cells to four-hour avasimibe treatment measured by markedly reduced pCREB and pS6 levels (Fig 4A). These findings implicated the MAPK pathway as a downstream target of avasimibe, which has been previously suggested.[15] To further investigate this, we performed mass cytometry screening of primary cells obtained from four BCR-ABL-independent resistant (RCML) and four imatinib-sensitive CML patients (SCML). We measured a number of phospho-markers (S1 Table) including five MAPK pathway proteins: p-p38, pCREB, pS6, pERK1/2, and pMAPKAP2. Phosphorylation of all MAPK proteins except pCREB were significantly reduced by imatinib in sensitive CML patients (Fig 4B). In contrast, no significant reduction in phosphorylation of the individual MAPK proteins was observed in resistant patients (Fig 4B). In addition, by performing a pooled analysis of the MAPK proteins, we determined that imatinib differentially affected sensitive but not resistant patients (p = .0013) (Fig 4B). With combination treatment, a significant difference in pERK levels was observed for resistant versus sensitive patients (Fig 4C), and a trend toward greater sensitivity was observed for pCREB, pS6, and p-p38 (but not pMAPKAP2) (Fig 4D, S5 Fig). These results collectively show that imatinib is sufficient to inhibit MAPK in sensitive patients, but combination therapy is capable and required to inhibit MAPK pathway proteins in resistant patients.

Due to the fact that K562R cells proliferate unhindered in lower concentrations of imatinib, we investigated the effect of lower-dose imatinib in combination with avasimibe on cell signaling in normal bone marrow as well as peripheral blood from a resistant (RCML1) patient and a sensitive (SCML4) patient, which were selected based upon sample availability. Mass cytometry analysis revealed that Lin^-^ CD34^+^ CD38^−^ cells in the imatinib-sensitive patient were profoundly sensitive to imatinib treatment, while combination treatment provided minimal additional effect on the levels of eight intracellular signaling markers (Fig 4E). Combination therapy also had minimal effect in normal bone marrow. The resistant patient’s cells also displayed sensitivity to imatinib as measured by pCRKL levels (canonical downstream target of BCR-ABL), suggesting that the resistance was indeed through BCR-ABL-independent mechanisms. However, in the resistant patient, imatinib treatment led to increased levels of p-p65/NFκB, p-p38/MAPK in hematopoietic stem and progenitor cells (Fig 4E and 4F, S7 Fig). Avasimibe treatment reversed the effect of imatinib, leading to reduced p-p65/NFκB and p-p38 levels in multiple progenitor populations (Fig 4E and 4F, S7 Fig). In the presence of imatinib, 49.7% of the cells were positive for p-p38 and/or p-p65/NFκB, while the addition of avasimbe to imatinib led to a reduction in the number of positive cells to 10.39% (Fig 4F). To understand the effect of treatment across the hematopoietic spectrum, viSNE[24], a dimensionality reduction tool, was used to demonstrate activation of p-p65/NFκB, p-p38, and pCREB broadly across the myeloid spectrum as a result of imatinib treatment, which was reversed by combination treatment (S6 and S7 Figs).

## Discussion

This study identifies CE accumulation as a unique feature of CML cells that could be a potential leukemia-specific target in future therapy. Constitutive BCR-ABL kinase activity was found to be sufficient and necessary to cause CE and LD accumulation. Prior clinical trials with the ACAT-1 inhibitor avasimibe to assess safety in atherosclerosis patients have demonstrated that this drug can be safely administered with minimal toxicity.[26] Our data suggest that avasimibe could specifically target cancer cells with minimal toxicity to blood cells lacking BCR-ABL.

A strong synergy of avasimibe and imatinib was found in BCR-ABL mutation-independent resistant K562R cells, but not in Ba/F3 BCR-ABL^T315I^ or naïve K562 cells. This suggests that avasimibe is targeting signaling pathways that are differentially activated in mutation-independent resistant CML compared to imatinib-naïve CML, or CML where resistance is a result of a BCR-ABL kinase-domain point mutation. However, it is worth noting that avasimibe monotherapy was sufficient to significantly inhibit Ba/F3^T315I^ and naïve K562 cell growth, which is consistent with their increased CE storage. It should be noted, however, that the data does not specifically support a role for CE in causing imatinib resistance, as both naïve K562 and K562R accumulate CE.

Mass cytometric analysis showed the effect of avasimibe on the MAPK pathway, which may contribute to the synergy of the two drugs specifically in resistant CML. MAPK has been shown to be a key regulator of BCR-ABL-independent imatinib resistance.[7] The mass cytometry results showed that imatinib alone is more potent in reducing MAPK protein phosphorylation in imatinib-sensitive patients than in resistant patients. This could be a result of MAPK activity in resistant patients being by driven by BCR-ABL independent mechanisms. In addition, our data showed that combination treatment had a stronger suppressive effect on the MAPK pathway in resistant patients, which could explain why K562R cells but not K562 cells respond synergistically to combination therapy.

Characterization of the mechanism of drug synergy by mass cytometry in a lower concentration of imatinib also revealed that the NFκB pathways may be another important regulator of BCR-ABL mutation-independent imatinib resistance. The NFκB pathway is known to have significant cross-talk with the MAPK pathway[27], which means that the NFκB effect is likely to be a result of MAPK activity. Thus, avasimibe could potentially resensitize resistant cells to imatinib treatment by inhibiting MAPK and NFκB activity while also causing free cholesterol mediated toxicity.[15] The synergistic inhibition of p-p38/MAPK and NFκB in IM-resistant patient samples by combination treatment provides a potential mechanism for our observed synergy in viability assays. Our data from the K562R xenograft mouse model further showed that inhibiting only BCR-ABL with imatinib or only MAPK/cholesterol esterification with avasimibe is not sufficient, but combination therapy significantly attenuated tumor growth. That finding is correlated with the fact that combination therapy was required to achieve decreased phosphorylation of all measured MAPK proteins in our mass cytometry experiments. Together, these results suggest that therapies targeting multiple drivers of leukemic proliferation may be needed to achieve a deeper treatment response in BCR-ABL mutation-independent resistant CML.

In summary, our data show that the combination of avasimibe and imatinib synergistically suppresses BCR-ABL mutation-independent imatinib-resistant CML proliferation by targeting cancer-specific CE accumulation, MAPK, and native BCR-ABL signaling. This drug combination is clinically relevant, as both of these drugs have been evaluated in clinical trials to assess their safety in humans. This approach also suggests the potential for combining relatively non-toxic metabolic inhibitors with existing therapies to overcome resistance in cancer cells.

## Supporting information

S1 Fig BCR-ABL induced LD and CE accumulation. (a) Raman spectral analysis of Ba/F3 BCR-ABL expressing cell lines. (b) Quantification of CE levels in Ba/F3 cell lines (c) SRS Imaging of 32D cells transduced with empty vector, BCR-ABL, BCR-ABL^T315I^, and BCR-ABL Kinase-Dead. Scale bar: 10 μm. (d) Cells were treated with avasimibe or DMSO for 48 hours, and the CE percentage was measured using Raman spectral analysis. (e) CE percentage from Raman spectral analysis was quantified.
(DOCX)

S2 Fig Combination Index of Imatinib and Avasimibe Combination Therapy. Combination index was calculated from the cell viability data in Fig 2C–2F and S3 Fig by the Chou-Talalay Method. CI < 1.0 indicates synergy.
(DOCX)

S3 Fig Combination treatment of Ba/F3 BCR-ABL^T315I^ cells does not yield a synergistic effect. (a) 3D Colormap Contour Plot of relative cell viability normalized to no inhibitor of Ba/F3 BCR-ABL^T315I^ after 72 hour treatment with a 1:10 constant combination ratio of imatinib to avasimibe measured by Cell Titer Glo. (b) Linear plot showing the relative cell viabilities of Ba/F3 BCR-ABL^T315I^ after 72 hour treatment with avasimibe alone, imatinib alone, and a 1:10 constant combination ratio of avasimibe to imatinib.
(DOCX)

S4 Fig Gating Hierarchy for isolating CD34(^+^) CD38(^−^) cells in each patient. Three examples of the gating used to isolate CD34^+^ CD38^-^ cells are shown here, from Fig 4E and 4F. The same gating was used for all patients in the study. Cells were isolated using bead normalization and DNA/length gating to specify single-cells. Cisplatin and Caspase3 were used as viability marker to ensure the health of the cells. Then, cells were gated to remove lymphoid cells, and CD34^+^ CD38^-^ cells were selected.
(DOCX)

S5 Fig Effect of avasimibe and imatinib combination treatment compared to imatinib alone. a-c) Fold change of 5μM imatinib + 10μM avasimibe compared to 5μM imatinib alone.
(DOCX)

S6 Fig Surface marker expression across the viSNE Map. viSNE plots are color coded by expression of surface markers, with red being the highest expression and blue being the lowest. viSNE plots represent all of the cells in a sample separated by phenotypic distance, or how variant the surface marker expression is. Similar cells will be grouped together, while highly different cells will be far apart.
(DOCX)

S7 Fig viSNE reveals imatinib response across myeloid spectrum. The top left plot shows the cell types in the viSNE map from the same experiment as panels (b) and (c), with each gate overlayed over the other and color-coded. The top right plot shows cell density in the viSNE map with red being the densest and blue being the least dense. Gating was done using the viSNE map. See S6 Fig for surface marker validation. The first set of four plots show p-p65/NFκB intensity across the four aforementioned conditions (top), the second set shows pCREB (middle), and the third set shows p-p38/MAPK (bottom). The maps are color-coded for marker signal intensity, with red being the maximum intensity.
(DOCX)

S1 Table Detailed information about the antibody panels used for the CyTOF experiments presented in this manuscript. (XLSX)
