# Supplementary material for: Cholesterol esterification inhibition and imatinib treatment synergistically inhibit growth of BCR-ABL mutation-independent resistant chronic myelogenous leukemia
Source: PLoS One. 2017 Jul 18;12(7):e0179558. doi: 10.1371/journal.pone.0179558 (PMC5515395; doi:10.1371/journal.pone.0179558)
Supplement: S6 Fig — viSNE plots are color coded by expression of surface markers, with red being the highest expression and blue being the lowest. viSNE plots represent all of the cells in a sample separated by phenotypic distance, or how variant the surface marker expression is. Similar cells will be grouped together, while highly different cells will be far apart. (DOCX) [file pone.0179558.s006.docx]

**S6 Fig.**
