# Supplementary material for: Cholesterol esterification inhibition and imatinib treatment synergistically inhibit growth of BCR-ABL mutation-independent resistant chronic myelogenous leukemia
Source: PLoS One. 2017 Jul 18;12(7):e0179558. doi: 10.1371/journal.pone.0179558 (PMC5515395; doi:10.1371/journal.pone.0179558)
Supplement: S7 Fig — The top left plot shows the cell types in the viSNE map from the same experiment as panels (b) and (c), with each gate overlayed over the other and color-coded. The top right plot shows cell density in the viSNE map with red being the densest and blue being the least dense. Gating was done using the viSNE map. See S6 Fig for surface marker validation. The first set of four plots show p-p65/NFκB intensity across the four aforementioned conditions (top), the second set shows pCREB (middle), and the third set shows p-p38/MAPK (bottom). The maps are color-coded for marker signal intensity, with red being the maximum intensity. (DOCX) [file pone.0179558.s007.docx]

**S7 Fig.**
